# Supplementary material for: Musical expertise generalizes to superior temporal scaling in a Morse code tapping task
Source: PLoS One. 2020 Jan 6;15(1):e0221000. doi: 10.1371/journal.pone.0221000 (PMC6944339; doi:10.1371/journal.pone.0221000)
Supplement: Attachment — Submitted filename: Review Slayton etal PLoS1.docx [file pone.0221000.s001.docx]

Review of „*Musical expertise generalizes to superior temporal scaling in a Morse code tapping task“*

by Slayton, Romero-Sosa, Stone, Buonomano & Viskontas

This paper describes a highly original experimental study which contrasts the timing performance of a simple rhythmic pattern (actually the word *time* in Morse code) by expert musicians vs. non-musicians. Participants practiced the corresponding temporal pattern at a fixed speed in 15 blocks of 15 trials each for four days, with detailed feedback about the accuracy of each production as compared to the target pattern. On day 5, participants were to produce the trained rhythmic pattern at each of three speeds: the original one, twice, or half as fast as the trained pattern. The goal of the study was to test the hypothesis that professional musicians are superior in *temporal scaling*, i.e., to transfer a rhythmic temporal pattern acquired at one fixed tempo to slower and to faster tempi. The main findings are summarized by the authors as

„Although there was no significant difference in performance at the end of training at the standard speed, musicians were significantly better at temporal scaling—i.e., at reproducing the learned Morse code pattern at faster and slower speeds. (…) Both musicians and non-musicians exhibited a *Weber-speed effect*, where absolute temporal precision sharpened when producing patterns at the faster speed.“

Not really unexpected..

My recommendation is to accept the paper after minor revision.

**Minor comments.**

Figures. The graphical quality is embarrassingly low. Here is an *enlarged* snapshot of task and stimuli:

Who you decode the details?

P. 3, 3rd par: „temporal variability“: as assessed by the standard deviation?

p. 4, top: Define NMRSE – not all readers will be familiar with it. Report whether you normalize by mean or by *range*.

p. 8, 2nd par: replace „time-independent“ by „tempo independent“.

p. 8, 2nd par: „Between each element that comprises a letter and the first element of another letter was a dot length pause, and between the last element of a letter and the first element of another letter was a dash-length pause.“ Why don’t you give just the sequence time-intervals to produce?
