# Supplementary material for: Musical expertise generalizes to superior temporal scaling in a Morse code tapping task
Source: PLoS One. 2020 Jan 6;15(1):e0221000. doi: 10.1371/journal.pone.0221000 (PMC6944339; doi:10.1371/journal.pone.0221000)
Supplement: Attachment — Submitted filename: Response to Reviewers.docx [file pone.0221000.s002.docx]

| Dean Buonomano, PhD  Brain Research Institute, UCLA  695 Charles Young Drive  Los Angeles, CA. 90025-1761 | DEPARTMENT OF NEUROBIOLOGY  DEPARTMENT OF PSYCHOLOGY  dbuono@ucla.edu  Phone: (310) 794-5009 |
| --- | --- |

November 4, 2019

Dear Dr. Grahn,

We would like to thank the reviewers for their helpful comments. We have addressed all of the points that the reviewers identified, and believe the manuscript has significantly improved as result of their suggestions.

We are appreciative of the opportunity to revise and improve our work, and we hope it is now suited for publication in **PLOS**.

Sincerely,

Matthew Slayton,

Dean Buonomano,

Indre Viskontas

**Detailed Responses to Reviewer Comments**

***Reviewer 1:***

We thank the reviewer for the assessment that “*The study investigated a novel and intriguing issue in the field of motor control”.*

*R1.1. First, the present Introduction is currently not clear for readers with respect to significance of the study. This can be largely improved by arguing the previous studies relevant to this study, which seems insufficient in the present manuscript. For example, temporal scaling has been already studied in musicians (e.g.* *Furuya and Soecthing 2012 J Neurophysiol), and therefore to argue them in the Introduction will make both novelty and significance of the study clearer. Similarly, neurophysiological studies such as bird songs have been also studied this issue extensively (Long and Fee 2008 Nature). Such neurophysiological studies will aid in deepening the understanding of what mechanisms can underlie the temporal scaling of sequential motor actions.*

We thank the reviewer for these suggestions and bringing these citations to our attention. We have now expanded the discussion of the motivation for and novelty of our experiments in the Introduction and Discussion. Additionally, we further explain that there is no contradiction with the Furuya and Soecthing paper because they were looking at *covariance* between striking and non-striking fingers when a pianist plays a particular note with one finger at different tempi, whereas we were looking at *accuracy* and *standard deviation* across successive trials.

*R1.2 it is better to provide a rationale of investigating the Morse code production task rather than piano playing, even though the latter is more naturalistic to the present participants. In general, a learning task used for sequential movements is a serial reaction time task (e.g. Karni et al. 1998 PNAS). As far as I know, the present motor task is not as frequently used as such a task, which makes me wonder a particular reason behind.*

We now elaborate in the Introduction of that we examine whether musical skill generalizes to a different domain, in this case a temporal pattern reproduction task. The advantage of the Morse Code task is that it is purely temporal, and thus allows us to potentially dissociate any temporal effects that are due to interactions between different finger use and time. Nevertheless, we recognize the importance of emphasizing how a purely temporal task may not generalize to a music-type task or a standard serial reaction-time task as used by Karni et al—which does not have an explicit temporal component. We clarify this point in the discussion.

*R.1.3 Related to this, the present Discussion can be largely improved by arguing an issue of signal-dependent noise in motor commands (Harris and Wolpert 1998 Nature) and/or Fitt's law. When moving faster, effects of this noise is increased, and vice versa. This neurophysiological mechanism will be crucial to shed light on improved temporal scaling with training.*

As suggested, we now also address the issue of the interaction between speed and noise in the Discussion. The relative increase of accuracy with increased speed is distinct from the phenomenon where there is a speed-accuracy trade-off.

*R1.4 There are some minor comments.

Abstract: page 8, line 7
What do you mean by "controls"?*

We have now clarified that the controls are non-musicians.

*R1.5 Introduction: page 9 second paragraph
While the authors state "It has also been shown that temporal precision can be improved by increasing movement speed (6).", this claim contradicts with the Fitts' Law. Please explain more.*

We now address this issue in the Discussion.

*R1.6 Figures are overall coarse in terms of resolution, which is better to be improved. Also I wonder if the color figures are really necessary.*

We have modified the figures and improved the resolution and removed unnecessary color use.

***Reviewer 2:***

We thank the reviewers view that *“This paper describes a highly original experimental study …”*

*R.2.1 Figures. The graphical quality is embarrassingly low. Here is an enlarged snapshot of task and stimuli:*

Fair point, and we apologize for the quality of the figures.

*R2.2 Who can decode the details?
P. 3, 3rd par: „temporal variability“: as assessed by the standard deviation?*

We now explicitly state that we were referring to standard deviation.

*R2.3 p. 4, top: Define NMRSE – not all readers will be familiar with it. Report whether you normalize by mean or by range.*

We have now better defined and described the NRMSE in the Methods section.

*R2.4 p. 8, 2nd par: replace „time-independent“ by „tempo independent“.*

Done.

*R2.5 p. 8, 2nd par: „Between each element that comprises a letter and the first element of another letter was a dot length pause, and between the last element of a letter and the first element of another letter was a dash-length pause.“ Why don’t you give just the sequence time-intervals to produce?*

We now also provide the absolute time of each press/release time of the target pattern.
